# Knowledge Regarding Ionizing Radiation Exposure Safety Among Orthopedic Surgeons at Hospitals in Al-Madinah

**DOI:** 10.7759/cureus.30738

**Published:** 2022-10-26

**Authors:** Yaser A Alshabi, Murad A Yasawy, Amin K Makhdoom, Rama A Kablaghli, Khalid S Alanazi, Siraj M Eid, Wafa M Imran

**Affiliations:** 1 Orthopaedics, King Fahad Hospital, Madinah, SAU; 2 Medicine and Surgery, Ibn Sina National College, Jeddah, SAU; 3 Sleep Medicine, King Abdulaziz University Hospital, Jeddah, SAU; 4 Emergency Medicine, King Abdullah University Hospital, Irbid, JOR; 5 Medicine and Surgery, Northern Border University, Arar, SAU; 6 Faculty of Medicine, King Abdulaziz University, Jeddah, SAU; 7 Medicine and Surgery, Shandong First Medical University, Tai'an, SAU

**Keywords:** floroscopy training, occupation safety, radiation exposure, ionizing radiation, orthopedic surgeons

## Abstract

Background and objective

The use of radiation imaging techniques in operation theaters is essential for numerous surgical procedures and patients’ overall well-being. Radiation imaging techniques enable the surgeon to have a real-time visualization of the anatomy and to perform operations with a greater chance of success, decrease rates of patient morbidity, and enable surgeons to obtain imaging records before the patient leaves the theater room. However, with the increased use of imaging techniques in orthopedic surgical operations, orthopedic surgeons are being exposed to higher levels of radiation, and hence they can be classified as a high-risk group for occupational radiation exposure. This study aimed to assess orthopedic surgeons' awareness and knowledge regarding radiation exposure safety.

Materials and methods

A questionnaire-based descriptive cross-sectional study was conducted from January to March 2022 to assess the knowledge regarding ionizing radiation exposure safety among orthopedic surgeons, including consultants, specialists, and residents, at both private and governmental hospitals in Al-Madinah city, Saudi Arabia. Ethical approval was obtained from the Ministry of Health (MOH) of Al-Madinah (approval number: H-03-l.l-084). The applied statistical tests were frequency and MCT tests for univariate variables while Chi-square was applied for bivariate variables. With a 95% confidence interval (CI), a p-value of more than 0.05 was used as the cut-off value for the significance level.

Results

A total of 57 surgeons participated in the study, of which 57.9% were exposed to radiation two to three times per week. Additionally, more than half of the physicians (66.7%) were not trained to use fluoroscopy (C-arm machine). Of note, 78.9% of orthopedic surgeons reported that they used the protective apron as protective equipment, while 17.5% of them used both a protective apron and thyroid shield. However, only less than half of the orthopedic surgeons (43.9%) in our study practiced radiation safety in the operating room.

Conclusion

Our study revealed a lack of knowledge and awareness related to ionizing radiation exposure safety among orthopedic surgeons in Al-Madinah city, Saudi Arabia.

## Introduction

The use of radiation imaging techniques in theater rooms is essential for numerous surgical procedures and patients’ overall well-being [1,2]. However, exposure to such radiation may have detrimental outcomes for the human body [1-3]. Radiation imaging techniques enable the surgeon to have a real-time visualization of the anatomy, which is highly useful for orthopedic surgeons as it allows them to perform their operations with a greater chance of success, less intraoperative time, decrease rates of patient morbidity, and enable surgeons to obtain imaging records before the patient leaves the theater room [3-5].

The increased use of imaging techniques in orthopedic surgical operations leads to increased exposure to radiation among orthopedic surgeons, and hence orthopedic surgeons may be classified as a high-risk group for occupational radiation exposure [3,5-7]. Despite being exposed to such radiation on a regular basis, the overall knowledge and awareness about radiation and safety protocols among orthopedic surgeons are lacking [2,7,8].

A study assessing the awareness of orthopedic surgeons using a survey asking physicians whether they received training with regard to radiation safety found that 87.3% had not received training in radiation safety despite 97% of the participants being exposed to fluoroscopy radiation at least once a week [2]. Another study concluded that the lack of knowledge might add to the radiation exposure concerns among orthopedic surgeons and there should be concerted efforts to raise knowledge and awareness about radiation exposure safety among surgeons who deal with ionizing exposure on a daily basis. Only 57.69% of the participants reported having some knowledge about radiation exposure [8].

Many research papers have shown that there is a link between exposure to ionizing radiation and many forms of cancers such as breast, thyroid, and lung [1,4,8], with the thyroid gland being particularly vulnerable to radiation exposure [9]. The harmful effects of regular exposure to ionizing radiation are not confined to cancerous growths, and cases of infertility, cataract, and skin changes have also been reported [2,4,7].

While a few studies on radiation safety have been conducted in Saudi Arabia, no studies have been done to specifically assess the knowledge and awareness concerning radiation exposure safety among orthopedic surgeons at Al-Madinah hospitals. Therefore, this study aimed to assess the awareness and knowledge of orthopedic surgeons at Al-Madinah hospitals regarding radiation exposure safety.

## Materials and methods

A questionnaire-based descriptive cross-sectional study was conducted from January to March 2022 to measure the knowledge related to Ionizing radiation exposure safety among orthopedic surgeons at both private and governmental hospitals in Al-Madinah city, Saudi Arabia. Ethical approval was obtained from the Ministry of Health (MOH) of Al-Madinah (approval number: H-03-l.l-084). A total of 57 surgeons participated in the study, including consultants, specialists, and residents. The response rate of the survey was 57%.

The questionnaire included a total of 19 questions, pertaining to the following aspects: professional rank, sex, institution, region, work experience, frequency of radiation exposure per week, previous experience in using fluoroscopy, sense of security and insecurity toward radiation exposure, usage of protective equipment, protective equipment check for effectiveness and exposure, usage of dosimeter, sending dosimeter results for measurement, whether radiation safety in the operating room is checked or not, presence of warning signs on the door in the operating room while performing fluoroscopy, concerns about radiation exposure, the person using fluoroscopy in the operating room, distance from fluoroscope while wearing protective equipment, the position of C-arm device while using it, and whether any previous training for radiation safety has been received. The validity of the questionnaire was determined by factor analysis and principal components [2].

Collected data were analyzed by using SPSS Statistics version 22 (IBM Corp., Armonk, NY). The applied statistical tests were frequency and MCT tests for univariate variables while the Chi-square test was employed for bivariate variables. With a 95% confidence interval (CI), a p-value of more than 0.05 was used as the cut-off value for the significance level.

## Results

As shown in Table 1, almost all the participants were male (87.7%, n=50), and 94.7% (n=54) were working in government hospitals; 43.9% (n=25) of the respondents were orthopedic surgical specialists while 35.1% and 21.1% (n=20, 12) were residents and consultants respectively. The mean work experience among the participants was 8.2 ±7.3 years.

**Table 1:** Demographic data

Rank/hospital type		Sex		Total
		Male	Female	
Resident	N	16	4	20
	%	32.00%	57.10%	35.10%
Specialist	N	22	3	25
	%	44.00%	42.90%	43.90%
Consultant	N	12	0	12
	%	24.00%	0.00%	21.10%
Total	N	50	7	57
	%	100.00%	100.00%	100.00%
Government	N	47	7	54
	%	94.00%	100.00%	94.70%
Private	N	3	0	3
	%	6.00%	0.00%	5.30%
Total	N	50	7	57
	%	100.00%	100.00%	100.00%

Regarding physician gender, rank, and how often they were exposed to ionizing radiation, as shown in Table 2, 57.9% of our physicians were exposed two to three times per week, while 31.6% were exposed six or more times per week. Notably, 60% (n=15) of the residents were exposed two to three times a week, while 36% (n=9) were exposed six or more times per week. We observed a statically significant difference in this regard (p=0.035). Male and female respondents were exposed two to three times a week at a rate of 56% and 71.4% respectively, with no statically significant difference (p=0.773).

**Table 2:** Association of gender and rank with exposure to ionizing radiation

Gender/rank		Exposure to ionizing radiation				Total	P-value
		Once a month	Once a week	2-3 times a week	6 or more times a week		
Male	N	1	5	28	16	50	0.773
	%	2.0%	10.0%	56.0%	32.0%	100.0%	
Female	N	0	0	5	2	7	
	%	0.0%	0.0%	71.4%	28.6%	100.0%	
Total	N	1	5	33	18	57	
	%	1.8%	8.8%	57.9%	31.6%	100.0%	
Resident	N	1	0	13	6	20	0.035
	%	5.0%	0.0%	65.0%	30.0%	100.0%	
Specialist	N	0	1	15	9	25	
	%	0.0%	4.0%	60.0%	36.0%	100.0%	
Consultant	N	0	4	5	3	12	
	%	0.0%	33.3%	41.7%	25.0%	100.0%	
Total	N	1	5	33	18	57	
	%	1.8%	8.8%	57.9%	31.6%	100.0%	

Table 3 shows that more than half (66.7%) of the physicians were not trained to use fluoroscopy (C-arm machine), with no significant difference in terms of either gender or ranking and training regarding the use of fluoroscopy (p=0.405, p=0.204 respectively).

**Table 3:** Association of gender and rank with being trained to use fluoroscopy (C-arm machine)

Gender/rank		Trained to use fluoroscopy		Total	P-value
		Yes	No		
Male	N	18	32	50	0.405
	%	36.0%	64.0%	100.0%	
Female	N	1	6	7	
	%	14.3%	85.7%	100.0%	
Total	N	19	38	57	
	%	33.3%	66.7%	100.0%	
Resident	N	4	16	20	0.204
	%	20.0%	80.0%	100.0%	
Specialist	N	9	16	25	
	%	36.0%	64.0%	100.0%	
Consultant	N	6	6	12	
	%	50.0%	50.0%	100.0%	
Total	N	19	38	57	
	%	33.3%	66.7%	100.0%	

Of note, 80.7% of the physicians in the cohort (80% of males and 85.7% of females) did not feel secure about fluoroscopy use and radiation exposure. As shown in Table 4, the study found that 78.9% of orthopedic surgeons used a protective apron as protective equipment, while 17.5% of the surgeons used both a protective apron and a thyroid shield. There was no significant association of either gender or professional rank with using any protective equipment (p=0.828, p=0.38 respectively).

**Table 4:** Association of gender and rank with the use of any protective equipment There were 5 options; 4 of them are presented in the table (no one chose radioprotective glasses)

Gender/rank		Use any protective equipment				Total	P-value
		I don't use any	Radioprotective gloves	Protective apron	Thyroid shield and protective apron		
Male	N	1	1	40	8	50	0.828
	%	2.0%	2.0%	80.0%	16.0%	100.0%	
Female	N	0	0	5	2	7	
	%	0.0%	0.0%	71.4%	28.6%	100.0%	
Total	N	1	1	45	10	57	
	%	1.8%	1.8%	78.9%	17.5%	100.0%	
Resident	N	0	1	16	3	20	0.38
	%	0.0%	5.0%	80.0%	15.0%	100.0%	
Specialist	N	0	0	21	4	25	
	%	0.0%	0.0%	84.0%	16.0%	100.0%	
Consultant	N	1	0	8	3	12	
	%	8.3%	0.0%	66.7%	25.0%	100.0%	
Total	N	1	1	45	10	57	
	%	1.8%	1.8%	78.9%	17.5%	100.0%	

Almost none of the participants (87.7%) checked for protective equipment effectiveness or expiration; 10.5% did not know that this was a factor, and only 1.8% checked before use; however, it had no statistically significant difference in terms of professional rank (p=0568). Similarly, almost none of the surgeons (94.7%) used a dosimeter for exposure, and only three of the specialists reported using it, of which two were sending it to the lab for measurements.

Less than half (43.9%) were practicing radiation safety in the operating room while 42.1% did not, with 14% reporting that they were not aware that this had to be done. Additionally, 57.9% of our participants denied the presence of warning signs on the door of the rooms when the fluoroscopy was used; 14% were unsure about the existence of such signs, while 28.1% confirmed the presence of the warning sign.

All of the surgeons were worried about radiation exposure; 84.2% were worried all the time, while 15.8% were worried some of the time. Of note, 90% of the residents were worried, while 76% and 91.7% of the specialists and consultants were worried respectively. However, there was no significant difference between the groups (p=0.321).

**Figure 1:**
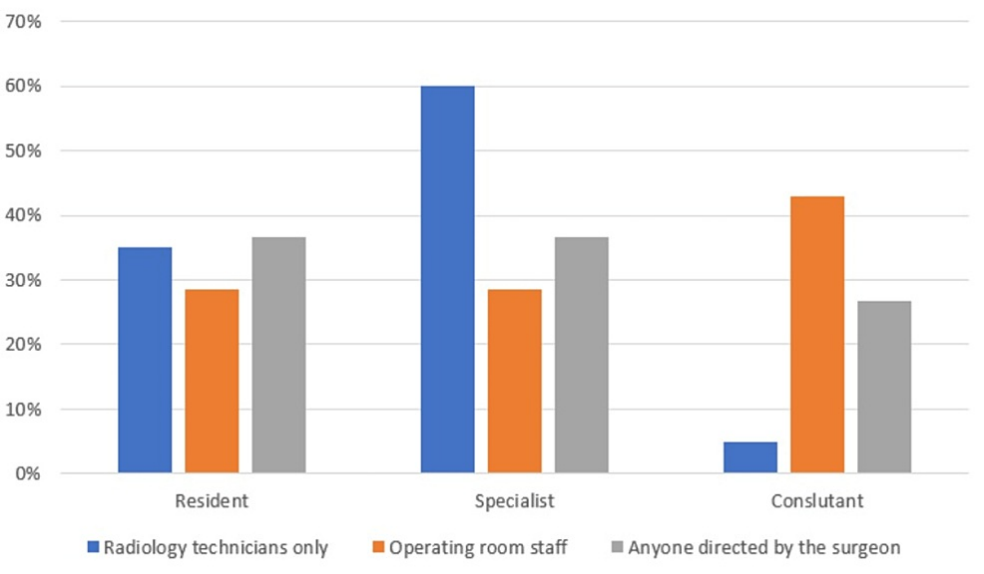
Professional rank and use of the fluoroscope in the operating room

As shown in Figure 1, 52.6% of the physicians stated that anyone directed by surgeons could use fluoroscopy in the operating room, and 57.9% of the physicians reported staying one to two steps away from the fluoroscope while using it, while 33.3% reported that distance from the equipment was not a cause for concern for them; lastly, 8.8% reported they stayed at least 3 meters away from it.

Regarding C-arm device position, 45.6% of the physicians put the X-ray tube (source) at the bottom and the X-ray receiver at the top; however, 31.6% put the X-ray receiver at the bottom and the X-ray tube (source) at the top, while 22.8% stated that this did not matter for them.

Finally, as shown in Table 5, 89.5% of the surgeons reported that they were not trained in radiation safety, and only 10.5% were trained. None of the consultants received this training, while 15% and 12% of the residents and specialists were trained in radiation safety, with no statically significant difference between the two groups (p=0.388).

**Table 5:** Professional rank and its association with being trained in radiation safety

Rank		Trained in radiation safety		Total	P-value
		Yes	No		
Resident	N	3	17	20	0.388
	%	15.0%	85.0%	100.0%	
Specialist	N	3	22	25	
	%	12.0%	88.0%	100.0%	
Consultant	N	0	12	12	
	%	0.0%	100.0%	100.0%	
Total	N	6	51	57	
	%	10.5%	89.5%	100.0%	

## Discussion

With the advancement in medical technology, the use of imaging modalities that utilize ionizing radiation has increased significantly; this could be attributed to the benefits they offer in terms of rapid and accurate diagnosing methods [2,3,5-10]. However, it is not without its adverse effects on the human body. Studies have shown that frequent and prolonged exposure to ionizing radiation can increase the risk of cancers as well as other diseases in various parts of the body [1,2,4,8-11]. In the present study, 80% of the male physicians and 85.7% of female physicians did not feel secure about exposure to ionizing radiation and the risk of these diseases.

Based on the guidelines by the National Council on Radiation Protection and Measurements (NCRP) and the International Commission on Radiological Protection (ICRP), the primary approach to minimizing exposure to ionizing radiation involves keeping exposure as low as reasonably achievable (ALARA). The main principle of ALARA involves decreasing the dose by adjusting the distance and time and utilizing shielding equipment; the results of our study revealed that many orthopedic surgeons at Al-Madinah hospitals lack knowledge and awareness regarding exposure to radiation safety and risks. Of note, 89.5% of physicians mentioned that they did have any prior training in radiation safety. A potential reason for the lack of awareness and practice could be the absence of an ionizing radiation safety training program during the residency years [2,11].

The thyroid gland is considered a very vulnerable organ, and it is susceptible to malignant changes with exposure to long-term ionizing radiation. Papillary thyroid carcinoma (PTC) accounts for around 85% of thyroid cancers and has been shown to have a very strong association with exposure to ionizing radiation [12,13]. It has also been observed that orthopedic surgeons are at a high risk of developing thyroid cancer [9]. The use of a thyroid protective apron has been shown to decrease the absorbed doses by the thyroid gland [9,10]. In our study, only 17.5% of the participants reported wearing a thyroid shield. A protective apron is a lead-containing vest that shields the trunk from radiation and is evaluated based on lead-equivalent thickness. A protective apron with a lead-equivalent thickness of 0.5 millimeters or higher will absorb 90-95% of radiation, provided that the apron has not been damaged [10,11]. Most of the participants (78.9%) wore a protective apron. However, almost none of the participants 87.7% checked the condition of their personal equipment. The eyes are also considered a sensitive organ that is vulnerable to radiation, and long-term exposure without proper protection to the eyes can cause cataracts [2,4,7,11]. Using protective eyewear is expected to reduce the radiation absorbed by up to 90% [11,14]. In our study, however, no one used protective eyewear.

The hands are the most exposed part of the body to radiation during operative procedures and sterile gloves do not provide effective protection. Hence, the use of radioprotective gloves is necessary to protect the hands from radiation [11]. In our study, only one participant (1.8%) reported using radioprotective gloves. A simple yet effective way of reducing radiation exposure is to simply stay as far away as possible from the source of radiation. This pertains to the rule of inverse-square law stating that the amount of radiation scattered is inversely proportional to the distance at which this radiation travels from the source [1,2,10,11]. In our study, only 8.8% of the participants stated that they stayed at least 3 meters away from the fluoroscope, while 57.9% reported they stayed one to two steps away from the device while using it.

Establishing proper and clear communication between the surgeon and the technician is vital as it follows one of the main principles of the ICRP: ensuring proper communication between the surgeon and the radiological technician reduces radiation exposure, operating time, and tension in the operating room [2,11,15]. Our data revealed that 52.6% of surgeons chose to allow anyone in the operating room to use the fluoroscope device. Such practices can cause an increased risk of radiation exposure.

Positioning the C-arm with the tube (source) under the operating table will significantly reduce the radiation scatter [1,10,11]; 45.6% of our participants stated that they positioned the tube (source) at the bottom of the operating table, while 54.4% of the participants noted that they did not care about the position.

Limitations

Every study encounters specific challenges and has its own limitations; in this study, our biggest challenge was the relatively small size of the sample since the number of orthopedics residents, specialists, and consultants at Al-Madinah hospitals is small. Hence, further studies with larger sample sizes are required to gain deeper insights into the subject. In addition, our response rate was 57%, and a higher rate would have been beneficial.

## Conclusions

Our study revealed an unfortunate lack of knowledge and awareness related to ionizing radiation exposure safety among orthopedic surgeons in Al-Madinah city, Saudi Arabia. The use of radioprotective gloves and protective eyewear among the participants was almost nonexistent. Measures should be taken to raise more awareness in terms of safety practices. All healthcare workers who are exposed to ionizing radiation should be aware of the risks and diseases associated with such exposure. Adhering to the main principles of the ALARA should be mandatory and a radiation safety training course should be introduced during the orthopedic residency.
